# Involvement of nucleoside diphosphate kinase b and elongation factor 2 in *Leishmania braziliensis* antimony resistance phenotype

**DOI:** 10.1186/s13071-016-1930-6

**Published:** 2016-12-13

**Authors:** Douglas S. Moreira, Silvane M. F. Murta

**Affiliations:** Centro de Pesquisas René Rachou CPqRR, Fundação Oswaldo Cruz - FIOCRUZ, Avenida Augusto de Lima 1715, Belo Horizonte, MG Brazil

**Keywords:** *Leishmania* spp., Nucleoside diphosphate kinase b, Elongation factor 2, Chemotherapy, Antimony resistance

## Abstract

**Background:**

Nucleoside diphosphate kinase b (NDKb) is responsible for nucleoside triphosphates synthesis and it has key role in the purine metabolism in trypanosomatid protozoans. Elongation factor 2 (EF2) is an important factor for protein synthesis. Recently, our phosphoproteomic analysis demonstrated that NDKb and EF2 proteins were phosphorylated and dephosphorylated in antimony (Sb^III^)-resistant *L. braziliensis* line compared to its Sb^III^-susceptible pair, respectively.

**Methods:**

In this study, the overexpression of *NDKb* and *EF2* genes in *L. braziliensis* and *L. infantum* was performed to investigate the contribution of these proteins in the Sb^III^-resistance phenotype. Furthermore, we examined the role of lamivudine on Sb^III^ susceptibility in clones that overexpress the *NDKb* gene, and the effect of EF2 kinase (EF2K) inhibitor on the growth of EF2-overexpressing parasites.

**Results:**

Western blot analysis demonstrated that NDKb and EF2 proteins are more and less expressed, respectively, in Sb^III^-resistant line of *L. braziliensis* than its wild-type (WTS) counterpart, corroborating our previous phosphoproteomic data. NDKb or EF2-overexpressing *L. braziliensis* lines were 1.6 to 2.1-fold more resistant to Sb^III^ than the untransfected WTS line. In contrast, no difference in Sb^III^ susceptibility was observed in *L. infantum* parasites overexpressing NDKb or EF2. Susceptibility assays showed that NDKb-overexpressing *L. braziliensis* lines presented elevated resistance to lamivudine, an antiviral agent, but it did not alter the leishmanicidal activity in association with Sb^III^. EF2-overexpressing *L. braziliensis* clone was slightly more resistant to EF2K inhibitor than the WTS line. Surprisingly, this inhibitor increased the antileishmanial effect of Sb^III^, suggesting that this association might be a valuable strategy for leishmaniasis chemotherapy.

**Conclusion:**

Our findings represent the first study of *NDKb* and *EF2* genes overexpression that demonstrates an increase of Sb^III^ resistance in *L. braziliensis* which can contribute to develop new strategies for leishmaniasis treatment.

## Background

Leishmaniasis refers to a disease complex caused by protozoan *Leishmania* parasites which are transmitted to humans by the bite of infected female phlebotomine sandflies. According to the World Health Organization (WHO), leishmaniasis is a neglected tropical disease that constitutes a public health problem in many developing countries of the Indian subcontinent, Latin America and East Africa [1]. Human leishmaniasis has an incidence of 1.2 million new cases annually, with an estimated population of 350 million at risk and a prevalence of 12 million cases [2]. Depending on genetic and environmental factors, the host immune response and mainly on *Leishmania* species involved, the disease can comprise three main clinical manifestations: cutaneous (CL), mucocutaneous (MCL) or visceral (VL) [3]. In the New World, *L.* (*Viannia*) *braziliensis* is the causative agent of CL and MCL, whereas *L.* (*Leishmania*) *infantum* [syn. *L.* (*L.*) *chagasi*] causes VL, which is lethal if not treated [4, 5].

Pentavalent antimonials (Sb^V^), such as sodium stibogluconate (Pentostam®) and meglumine antimoniate (Glucantime®), remain the first-line of treatment against all forms of the disease for more than 70 years especially in developing countries [6]. Despite the mechanism of antimony action has not been completely elucidated, studies suggest that Sb^V^ is reduced to trivalent form (Sb^III^) that is active against amastigote and promastigote forms of *Leishmania* [7]. Earlier reports have indicated that antimonials inhibit fatty acid β-oxidation and glycolysis [8], and cause perturbations in the thiol redox potential, which would drive to parasite death by oxidative stress [9]. Furthermore, it has been suggested that antimony can kill the parasite by an apoptosis process resulting in DNA fragmentation and externalization of phosphatidylserine outside of *Leishmania* [10, 11].

The emergence and spread of resistance to antimony is significant in determined regions, such as Bihar state in India where over 60% of VL patients do not respond to the traditional therapy using antimonials [12]. Recent studies have demonstrated several mechanisms in *Leishmania* species implicated in resistance to these compounds. Thus, antimony resistance constitutes a multifactorial process and involves at least some of following aspects. A decrease in rate of reduction from pentavalent to trivalent form or loss of reductase activity may lead to drug resistance [13, 14]. Lower expression of AQP1 (aquaglyceroporin), which is involved in Sb^III^ uptake into parasite, was also observed in resistant mutants [15, 16]. Increased levels of intracellular thiols were found in cells selected for resistance to Sb^III^ [17] as well as in unresponsive clinical isolates [18–20]. MRPA (multidrug resistance associated protein A) transporter confers resistance by sequestering thiol-Sb conjugates into an intracellular vacuole which removes the drug from the cytoplasm of the parasite [21]. An increase of PGP (phosphoglycoprotein) expression was described in *Leishmania* resistant to antimonials [22], suggesting that this protein mediates the efflux of these drugs from the parasite. Moreover, other mechanisms can also contribute to the antimony resistance phenotype in *Leishmania*.

Nucleoside diphosphate kinase b (NDKb), a NDK family member, is ubiquitous enzyme that is crucial to transfer phosphate group from a nucleoside triphosphate (NTP) to a nucleoside diphosphate (NDP), using a ping-pong mechanism that involves a phosphohistidine intermediate [23, 24]. The whole reaction is described as follows [25]:

$$ \mathrm{N}\mathrm{D}\mathrm{K}+{\mathrm{N}}_1\mathrm{T}\mathrm{P}\ \leftrightarrow\ \mathrm{N}\mathrm{D}\mathrm{K}\hbox{-} \mathrm{P} + {\mathrm{N}}_1\mathrm{D}\mathrm{P}\ \leftrightarrow\ \mathrm{N}\mathrm{D}\mathrm{K}\hbox{-} \mathrm{P} + {\mathrm{N}}_2\mathrm{D}\mathrm{P}\ \leftrightarrow\ \mathrm{N}\mathrm{D}\mathrm{K} + {\mathrm{N}}_2\mathrm{T}\mathrm{P} $$

NDKs play pivotal roles in different organisms, such as in bacterial pathogenesis [26], regulation of gene expression in cells of mammals [27] and participation in the purine salvage pathways of protozoan parasites [28]. NTP is a precursor for DNA and RNA synthesis, evidencing that NDK is also an essential enzyme for all cellular processes involving nucleic acids in distinct species of organisms [24]. Unlike mammals, *Leishmania* is not able to synthesize purines by means of a *de novo* mechanism and thus depend upon the host for survival [29]. Kolli et al. [30] showed that *Leishmania*-released NDK avoids ATP-mediated lysis of macrophages, keeping the integrity of host cells to the advantage of the parasite. Therefore, NDKb can be considered an interesting target for drug discovery for chemotherapy of leishmaniasis.

The regulation of protein synthesis in eukaryotic cells occurs via both initiation and elongation levels. This process uses significant quantity of cellular energy, and the vast majority of this is consumed in elongation [31]. Elongation factor 2 (EF2) codifies a member of the GTP-binding translation elongation factor family and it is a relevant factor for production of proteins. EF2, which is a ubiquitous enzyme, makes the GTP-dependent translocation of the aminoacyl-tRNA from the A-site to the P-site of the ribosome [32]. EF2 protein was found with increased abundance in promastigote forms of *L. panamensis* resistant to Sb^III^ [33].

Considering the multiplicity of antimony resistance mechanisms, our knowledge about them in New World *Leishmania* species is far from being fully elucidated. Recently, our phosphoproteomic analysis demonstrated that the nucleoside diphosphate kinase b (NDKb) and elongation factor 2 (EF2) proteins were phosphorylated and dephosphorylated, respectively, in Sb^III^-resistant *L. braziliensis* line compared to its wild-type (WTS) counterpart [34]. In this study, *NDKb* and *EF2* genes were transfected in Sb^III^-susceptible lines of *L. braziliensis* and *L. infantum* to determine whether the overexpression of these proteins contributes to antimony resistance phenotype in these parasites. Moreover, we investigated the role of lamivudine on Sb^III^ susceptibility in NDKb-overexpressing clones, and the effect of EF2 kinase (EF2K) inhibitor on the growth of EF2-overexpressing parasites.

## Methods

### *Leishmania* spp. cultures

We used promastigote forms of *L. braziliensis* (MHOM/BR/75/M2904) and *L. infantum* (MHOM/BR/74/PP75) in our study. The antimony-resistant lines were previously selected in vitro to potassium antimonyl tartrate (Sb^III^) (C_8_H_4_K_2_O_12_Sb_2_.3H_2_O) by step-wise drug pressure and their resistance indices were 20-fold and 4-fold higher than those of their wild-type counterparts, respectively [35]. Parasites were grown at 26 °C in M199 medium supplemented with 2 mM L-glutamine, 5 μg/ml hemin, 50 μg/ml streptomycin, 2 μg/ml biopterin, 1 μg/ml biotin, 40 mM HEPES pH 7.4, 500 U penicillin and 10% v/v heat-inactivated fetal calf serum [35]. These parasites were harvested in the logarithmic growth phase to perform all analyses.

### Generation of NDKb and EF2 overexpressing lines

A 456 bp fragment corresponding to *NDKb* encoding region (TriTrypDB accession number LbrM.32.3210) was amplified with *Pfx* DNA polymerase (Invitrogen) from *L. braziliensis* genomic DNA using the forward primer: 5′-TGG ATC CCC ACC ATG TCC TCC GAG CGC ACT TT-3′ and the reverse primer: 5′-TTG GAT CCC TAT TCG TAG ATC TGG CAA GCG G-3′. Other 2,538 bp fragment corresponding to *EF2* encoding region (TriTrypDB accession number LbrM.35.0270) was also amplified with the enzyme cited above using *L. braziliensis* genomic DNA and the primers forward: 5′-TGG ATC CCC ACC ATG GTG AAC TTT ACC GTC GAT CAG-3′ and reverse: 5′-TTG GAT CCT TAC AAT TTA TCC ATG AAC TGG TCC A-3′. The underlined sequences correspond to *Bam*HI restriction site. The obtained PCR products were cloned into the pGEM-T Easy® vector (Promega, Madison, WI, USA) and subsequently submitted to sequencing reaction for confirmation of correct sequence. All constructs were sequenced in an ABI 3130 (Applied Biosystems, Foster City, CA, USA). The pGEM-NDKb and pGEM-EF2 constructs were restricted with *Bam*HI and the fragments released were subcloned into the dephosphorylated pIR1BSD expression vector (kindly provided by Dr. Stephen Beverley, Washington University, USA). To confirm the correct direction of cloning, the constructs were then digested with *Hind*III and *Sma*I releasing fragments that confirmed the sense direction of the genes *NDKb* and *EF2*, respectively. Thus, the constructs pIR1BSD (empty vector), pIR1BSD-NDKb and pIR1BSD-EF2 were linearized by *Swa*I digestion and electroporated into wild-type *L. braziliensis* and *L. infantum* lines using a GenePulser XCell electroporation system (Bio-Rad, Hercules, CA, USA). This allowed integration of the vector into the 18S ribosomal DNA small subunit locus [36]. Colonies were obtained following plating on semisolid M199 medium containing blasticidin (BSD) (10 μg/ml). After 1–2 weeks, clonal lines were selected and the presence of constructs was confirmed by PCR tests using genomic DNA with primers specific for the BSD marker.

### Protein levels

Western blot assays were carried out for investigating the expression level of NDKb and EF2 proteins in the transfected parasites and in the Sb^III^-resistant and -susceptible lines of *L. braziliensis* and *L. infantum*. Total proteins from these parasites were extracted according to the protocol previously described [37]. Subsequently, 20 μg from each sample were separated by electrophoresis on 12% SDS-polyacrylamide gel and transferred onto nitrocellulose membranes (Bio-Rad, Hercules, CA, USA). They were blocked, washed and probed with rabbit polyclonal anti-NDKb (1:200) (Abcam, Cambridge, UK, #ab154274) or rabbit monoclonal anti-EF2 (1:200) (Abcam, Cambridge, UK, #ab75748) antibodies, during 12 h at 4 °C in the blocking solution. According to manufacturer specifications, the immunogen of the first antibody is a recombinant fragment corresponding to a region within amino acids 1–105 of human NDKb, while the immunogen of the second antibody is a synthetic peptide corresponding to residues on the C terminal of human EF2 (Abcam, Cambridge, UK). The blots were washed twice and incubated with horseradish peroxidase-conjugated anti-rabbit IgG (1:5,000) (GE Healthcare) for 1 h at room temperature. After incubation, the membranes were washed, incubated with ECL Plus chemiluminescent substrate (GE Healthcare) and revealed by ImageQuant LAS 4000 (GE Healthcare). To confirm equivalent loading, SDS-PAGE containing the samples were stained with Coomassie blue. Furthermore, the blots were normalized using the antibody monoclonal anti-α-tubulin (1:15,000) (Sigma, St. Louis, USA). The intensity of the bands was analyzed using the software GelAnalyzer 2010 (gelanalyzer.com).

### Susceptibility assays of *Leishmania* spp. clonal lines to Sb^III^ and H_2_O_2_

Promastigotes of wild-type *L. braziliensis* and *L. infantum* clonal lines non-transfected or transfected with the constructs pIR1BSD (empty vector), pIR1BSD-NDKb or pIR1BSD-EF2 were submitted to Sb^III^ (Sigma-Aldrich, St. Louis, MO, USA) susceptibility tests. The susceptibility to hydrogen peroxide (H_2_O_2_) was also evaluated in the parasites transfected with the *NDKb* gene. Parasites were incubated in M199 medium at 2 × 10^6^ cells/ml into 24-well plates in the absence or presence of several concentrations of Sb^III^ (1.17 to 599.04 μM which corresponds to 0.00078125 to 0.4 mg/ml) or H_2_O_2_ (100 to 350 μM) for 48 h. The effective concentration required to decrease growth by 50% (EC_50_) was determined using a model Z1 Coulter Counter (Beckman Coulter, Fullerton, CA, USA). EC_50_ values were determined from at least three independent measurements performed in triplicate, using the linear interpolation method [38].

### Susceptibility assays of *L. braziliensis* lines to lamivudine and EF2K inhibitor and competition assays

We determined the susceptibility of *L. braziliensis* lines to lamivudine (2′,3′-dideoxy-3′-thiacytidine - C_8_H_11_N_3_O_3_S) (Globe Química) (kindly provided by Dr. Juliana Medeiros and Dr. Ana Cláudia Tavares, Farmanguinhos/FIOCRUZ, Brazil) and 7-Amino-1-cyclopropyl-3-ethyl-1,2,3,4-tetrahydro-2,4-dioxopyrido [2,3-*d*] pyrimidine-6-carboxamide (C_13_H_15_N_5_O_3_) (Tocris Bioscience, A484954) which are inhibitors for the enzymes NDKb and elongation factor 2 kinase (EF2K), respectively. EC_50_ of these inhibitors for wild-type *L. braziliensis* line and parasites that overexpress the *NDKb* or *EF2* genes was determined to be used in competition tests with Sb^III^. The cultures (2 × 10^6^ cells/ml) were incubated in the absence or presence of various concentrations of lamivudine (125 to 10,000 μM) or EF2K inhibitor (50 to 300 μM) into 24-well plates during 48 h. After, the percentage of relative growth was determined by automated cell counting. Competition assays were performed to investigate the leishmanicidal activity of the inhibitors cited above in association with Sb^III^. In these experiments, 2 × 10^6^ parasites/ml were seeded into 24-well cell culture plates containing medium M199. Subsequently, the EC_50_ of lamivudine and EF2K inhibitor were added concomitantly with Sb^III^ EC_50_ (Table 1), followed by incubation for 48 h. The percentage of relative growth was determined by automated cell counting using a Z1 Coulter Counter.

**Table 1:** EC_50_ of Sb^III^, lamivudine, EF2K inhibitor and hydrogen peroxide and corresponding RI for WTS, NDKb- or EF2-overexpressing *L. braziliensis* lines. EC_50_ represents effective concentration required to decrease growth by 50%. The values were determined from at least three independent experiments performed in triplicate, using the linear interpolation method [38]

Parasites	Sb^III^		Lamivudine		EF2K inhibitor		Hydrogen peroxide	
	EC_50_ (μM)	RI	EC_50_ (μM)	RI	EC_50_ (μM)	RI	EC_50_ (μM)	RI
LbWTS	7	–	766	–	173	–	213	–
LbpIR1BSD	8	–	733	–	–	–	207	–
LbNDKb clone 4	12	1.7	2,110	2.8	–	–	207	1.0
LbNDKb clone 9	15	2.1	2,014	2.6	–	–	207	1.0
LbEF2 clone 9	14	2.0	–	–	211	1.2	–	–
LbEF2 clone 12	11	1.6	–	–	–	–	–	–

*Chemical formulae and abbreviations*: Sb^III^ (potassium antimonyl tartrate); lamivudine (C_8_H_11_N_3_O_3_S); EF2K inhibitor (C_13_H_15_N_5_O_3_); hydrogen peroxide (H_2_O_2_); *WTS* wild-type susceptible, *Lb L.* (*V*.) *braziliensis*, *pIR1BSD* expression vector, *NDKb* nucleoside diphosphate kinase b, *EF2* elongation factor 2, *EF2K* elongation factor 2 kinase, *RI* resistance index

### Statistical analysis

Data were analyzed by Student’s *t*-test performed using the software GraphPad Prism 5.0. A *P-*value less than 0.05 was considered statistically significant.

## Results

### Expression levels of NDKb and EF2 proteins in *Leishmania* lines

**Fig. 1:**
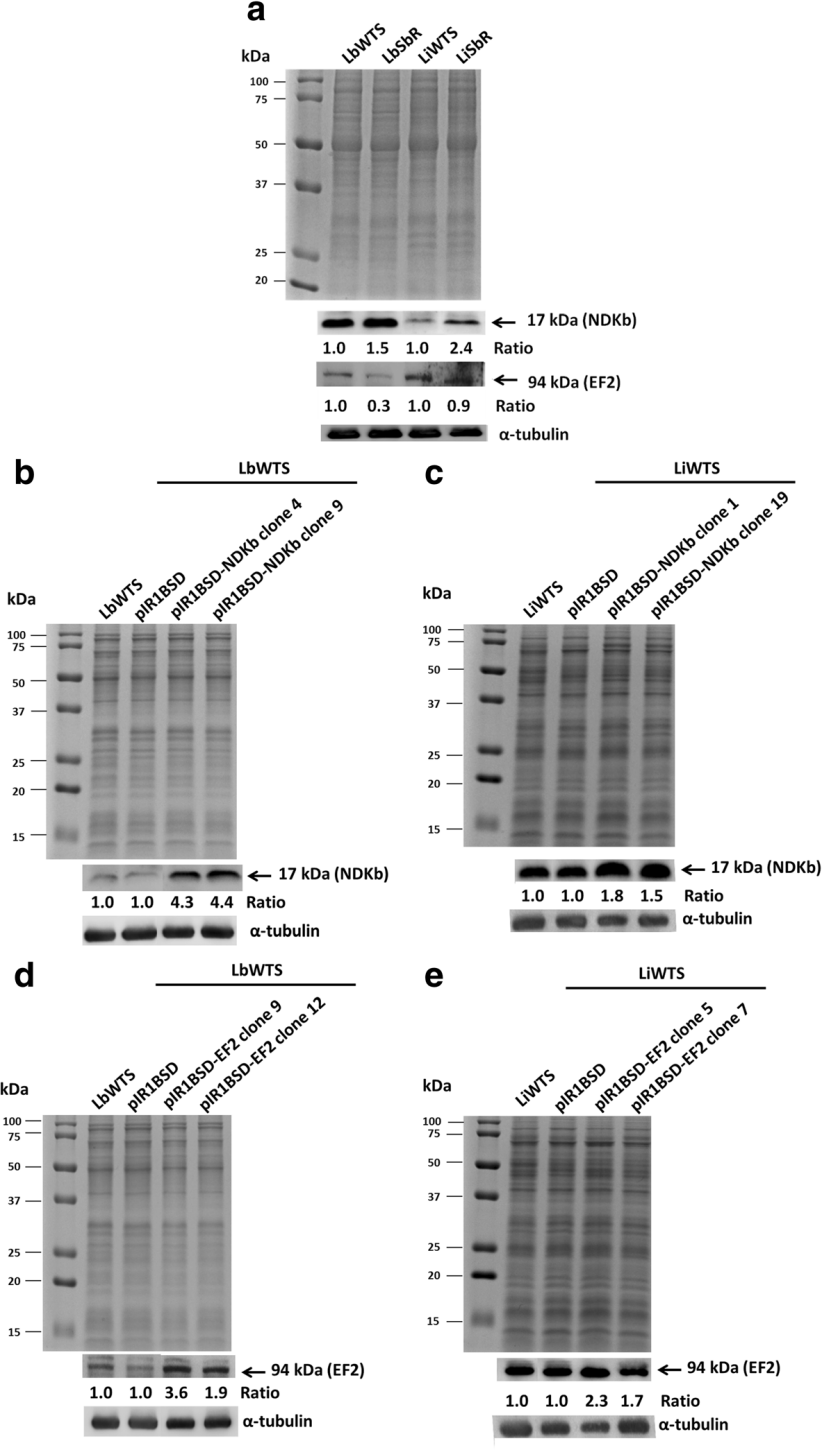
NDKb and EF2 expression levels in wild-type (WTS), Sb^III^-resistant (SbR) and clonal lines from *L. braziliensis* and *L. infantum* untransfected or transfected with the constructs pIR1BSD (empty vector), pIR1BSD-NDKb or pIR1BSD-EF2. Total proteins (20 μg) were separated by electrophoresis on 12% SDS-polyacrylamide gel and transferred onto nitrocellulose membranes. The profiles of total proteins stained with Coomassie blue are shown. The blots were probed with rabbit polyclonal anti-NDKb (1:200) (**a**, **b** and **c**) or rabbit monoclonal anti-EF2 (1:200) (**a**, **d** and **e**) antibodies and developed using ECL Plus kit. All membranes were incubated with the anti-α-tubulin monoclonal (1:15,000) antibody for normalization of the results. Quantification of the bands was done by densitometric analysis using the software GelAnalyzer 2010. The figure is representative of all results obtained from two different biological replicates of each sample. The values of each replicate were used to calculate the averages and for determination of the ratios presented for each protein analyzed

We determined the expression levels of the proteins NDKb and EF2 in the antimony-susceptible and antimony-resistant *L. braziliensis* and *L. infantum* lines by Western blot analysis using polyclonal anti-NDKb and monoclonal anti-EF2 antibodies, respectively. These antibodies are specific for mammalian proteins. It is important to highlight that the identity between the NDKb and EF2 amino acid sequences of *L. braziliensis* compared to mammalian was 66 and 61%, respectively (data not shown). Western blot results revealed that these antibodies recognized polypeptides of 17 kDa and 94 kDa in all *Leishmania* samples analyzed which correspond to the expected size of NDKb and EF2 proteins, respectively (Fig. 1a). The membranes were incubated with the monoclonal anti-α-tubulin antibody for normalization of the results (Fig. 1a). The expression level of NDKb protein was 1.5-fold and 2.4-fold higher in the Sb^III^-resistant *Leishmania* spp. lines (LbSbR and LiSbR) in comparison with their respective wild-type lines (LbWTS and LiWTS) (Fig. 1a). Regarding expression level of EF2 protein, the results demonstrated that this protein was approximately 3-fold lower in the Sb^III^-resistant *L. braziliensis* line when compared to its wild-type counterpart LbWTS. On the other hand, the EF2 protein presented the same level of expression between the LiWTS and LiSbR lines (Fig. 1a).

### Overexpression of *NDKb* and *EF2* genes in *L. braziliensis* and *L. infantum*

Wild-type *L. braziliensis* and *L. infantum* lines were transfected with the constructs containing the *NDKb* or *EF2* genes (pIR1BSD-NDKb or pIR1BSD-EF2) and empty vector (pIR1BSD) to generate parasites overexpressing the enzymes NDKb and EF2. Linearization of this vector allowed integration of the constructs into the ribosomal small subunit locus [36]. To confirm the transfection, genomic DNA from the transfected clones was subjected to PCR assays using specific primers for the *BSD* gene, which confers resistance to blasticidin. It was observed that all blasticidin-resistant clones showed a fragment of 399 bp, which corresponds to BSD marker (data not shown). These clones were subjected to Western blot assays in order to evaluate if the NDKb and EF2 enzymes were overexpressed. Our results showed that the level of NDKb protein expression was 1.5 to 4.4-fold higher in the transfected clones from *L. braziliensis* and *L. infantum* lines than in the non-transfected or transfected with empty vector (controls) (Fig. 1b, c). Furthermore, our analysis also demonstrated that the expression level of EF2 protein was increased 1.7 to 3.6-fold in the transfected clones from these both wild-type *Leishmania* lines when compared to their respective controls (Fig. 1d, e).

### Susceptibility of NDKb and EF2 overexpressing *Leishmania* spp. lines to Sb^III^

**Fig. 2:**
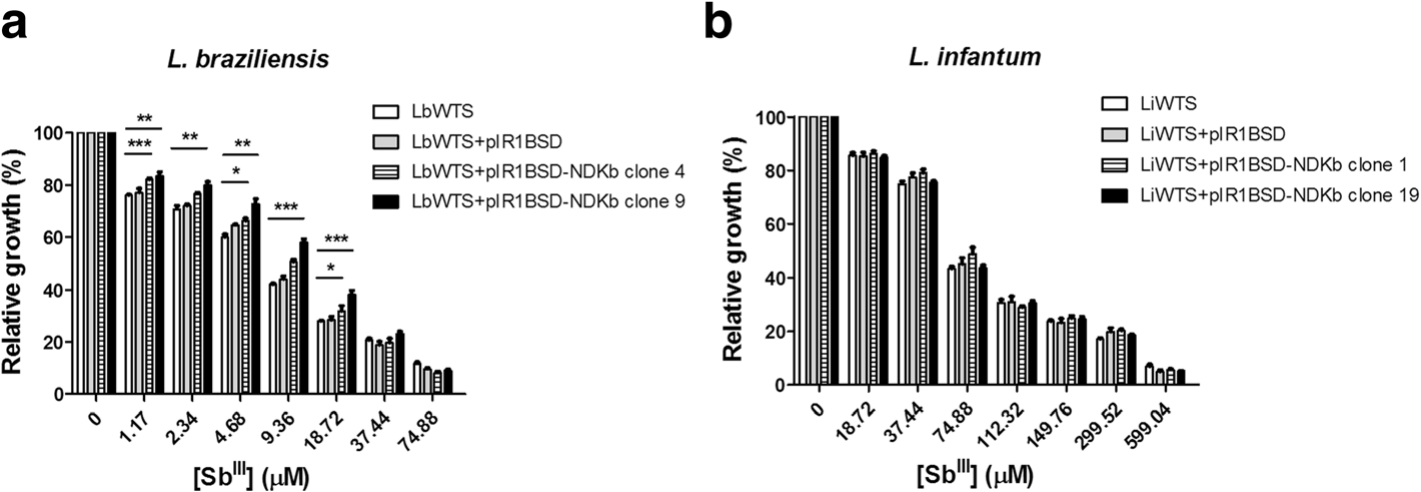
Sb^III^ susceptibility assay of clonal lines from *L. braziliensis* (**a**) and *L. infantum* (**b**) non-transfected or transfected with the constructs pIR1BSD or pIR1BSD-NDKb. Parasites were incubated in M199 medium in the absence or presence of different concentrations of Sb^III^ (1.17 to 599.04 μM) for 48 h and the percentage of relative growth was determined using a Z1 Coulter Counter. Mean values ± standard deviations from three independent experiments in triplicate are shown. Statistical analysis was carried out using Student’s *t-*test. Statistically different values are highlighted as follows: *P < 0.05; **P < 0.01; ***P < 0.001. Pairwise comparisons (**a**): **1.17 μM:** LbWTS *vs* LbNDKb clone 4 (*t*_(5)_ = 9.47, *P* = 0.0002); LbWTS *vs* LbNDKb clone 9 (*t*_(9)_ = 3.36, *P* = 0.0084); **2.34 μM**: LbWTS *vs* LbNDKb clone 4 (*t*_(5)_ = 4.3, *P* = 0.0077); LbWTS *vs* LbNDKb clone 9 (*t*_(8)_ = 3.74, *P* = 0.0057); **4.68 μM:** LbWTS *vs* LbNDKb clone 4 (*t*_(4)_ = 4.02, *P* = 0.0159); LbWTS *vs* LbNDKb clone 9 (*t*_(5)_ = 4.99, *P* = 0.0042); **9.36 μM:** LbWTS *vs* LbNDKb clone 4 (*t*_(6)_ = 8.39, *P* = 0.0002); LbWTS *vs* LbNDKb clone 9 (*t*_(8)_ = 12.50, *P* < 0.0001); **18.72 μM:** LbWTS *vs* LbNDKb clone 4 (*t*_(8)_ = 2.88, *P* = 0.0206); LbWTS *vs* LbNDKb clone 9 (*t*_(10)_ = 7.36, *P* < 0.0001)

We also investigated whether the overexpression of *NDKb* and *EF2* genes contributes to antimony resistance phenotype in *Leishmania*. For this, clonal lines from *L. braziliensis* and *L. infantum* transfected with the constructs pIR1BSD (empty vector), pIR1BSD-NDKb or pIR1BSD-EF2 and untransfected parasites were incubated with different Sb^III^ concentrations. The EC_50_ was determined by counting of parasites number grown in the absence or presence of this drug. The results showed that the Sb^III^ EC_50_ of untransfected *L. braziliensis* line was 7 μM. On the other hand, the clones 4 and 9 that overexpress NDKb in this *Leishmania* species presented EC_50_ of 12 μM and 15 μM, demonstrating an increase of 1.7 and 2.1-fold in the Sb^III^ resistance index of these clones, respectively (Fig. 2a) (Table 1). In contrast, NDKb-overexpressing *L. infantum* lines did not show an increase in resistance towards Sb^III^. The results revealed that the Sb^III^ EC_50_ of wild-type *L. infantum* line was 67 μM, and the NDKb overexpressing clones 1 and 19 was 81 and 73 μM, respectively (Fig. 2b).

**Fig. 3:**
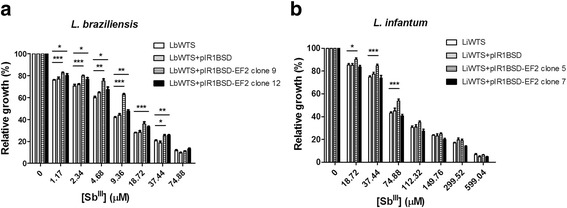
Sb^III^ susceptibility assay of clonal lines from *L. braziliensis* (**a**) and *L. infantum* (**b**) non-transfected or transfected with the constructs pIR1BSD or pIR1BSD-EF2. Parasites were incubated in M199 medium in the absence or presence of different concentrations of Sb^III^ (1.17 to 599.04 μM) for 48 h and the percentage of relative growth was determined using a Z1 Coulter Counter. Mean values ± standard deviations from three independent experiments in triplicate are shown. Statistical analysis was carried out using Student’s *t*-test. Statistically different values are highlighted as follows: *P < 0.05; **P < 0.01; ***P < 0.001. Pairwise comparisons (**a**): **1.17 μM:** LbWTS *vs* LbEF2 clone 9 (t_(9)_ = 6.5, *P* = 0.0001); LbWTS *vs* LbEF2 clone 12 (t_(5)_ = 2.84, *P* = 0.0361); **2.34 μM**: LbWTS *vs* LbEF2 clone 9 (t_(7)_ = 6.95, *P* = 0.0002); LbWTS *vs* LbEF2 clone 12 (t_(4)_ = 2.87, *P* = 0.0455); **4.68 μM:** LbWTS *vs* LbEF2 clone 9 (t_(5)_ = 6.04, *P* = 0.0018); LbWTS *vs* LbEF2 clone 12 (t_(4)_ = 2.87, *P* = 0.0453); **9.36 μM:** LbWTS *vs* LbEF2 clone 9 (t_(7)_ = 22.18, *P* < 0.0001); LbWTS *vs* LbEF2 clone 12 (t_(8)_ = 4.48, *P* = 0.0021); **18.72 μM:** LbWTS *vs* LbEF2 clone 9 (t_(11)_ = 4.74, *P* = 0.0006); LbWTS vs. LbEF2 clone 12 (t_(9)_ = 7.65, *P* < 0.0001); **37.44 μM:** LbWTS *vs* LbEF2 clone 9 (t_(5)_ = 2.78, *P* = 0.0391); LbWTS *vs* LbEF2 clone 12 (t_(5)_ = 5.1, *P* = 0.0038). (**b**) **18.72 μM:** LiWTS *vs* LiEF2 clone 5 (t_(9)_ = 2.67, *P* = 0.0257); **37.44 μM:** LiWTS *vs* LiEF2 clone 5 (t_(8)_ = 6.14, *P* = 0.0003); **74.88 μM:** LiWTS *vs* LiEF2 clone 5 (t_(9)_ = 4.9, *P* = 0.0008)

Regarding *EF2* gene, we observed that Sb^III^ EC_50_ of EF2-overexpressing clones 9 and 12 in *L. braziliensis* was 14 μM and 11 μM, showing an increase of 2.0 and 1.6-fold in the resistance index of Sb^III^ of these clones, respectively (Fig. 3a) (Table 1). On the other hand, EF2-overexpressing *L. infantum* lines did not alter the Sb^III^ susceptibility. The Sb^III^ EC_50_ of EF2-overexpressing clones 5 and 7 was 80 μM and 67 μM, respectively (Fig. 3b).

It is important to mention that NDKb- or EF2-overexpressing parasites presented similar growth curves when compared with their respective controls for both *Leishmania* species (data not shown). In the Sb^III^ susceptibility assays, we observed gradual reduction in the growth of NDKb- or EF2-overexpressing parasites of *L. braziliensis* with increasing drug concentrations. In addition, we observed that the overexpressors of NDKb or EF2 were more resistant to Sb^III^ until concentrations near to EC_50_ of these clones. In higher antimony concentrations, these parasites presented similar growth to their controls. We also observed that the vector used in our assays did not interfere in the Sb^III^ susceptibility since no difference in Sb^III^ EC_50_ was observed between parasites untransfected and transfected with empty vector.

### Effect of lamivudine on the growth of wild-type and NDKb-overexpressing *L. braziliensis* lines

**Fig. 4:**
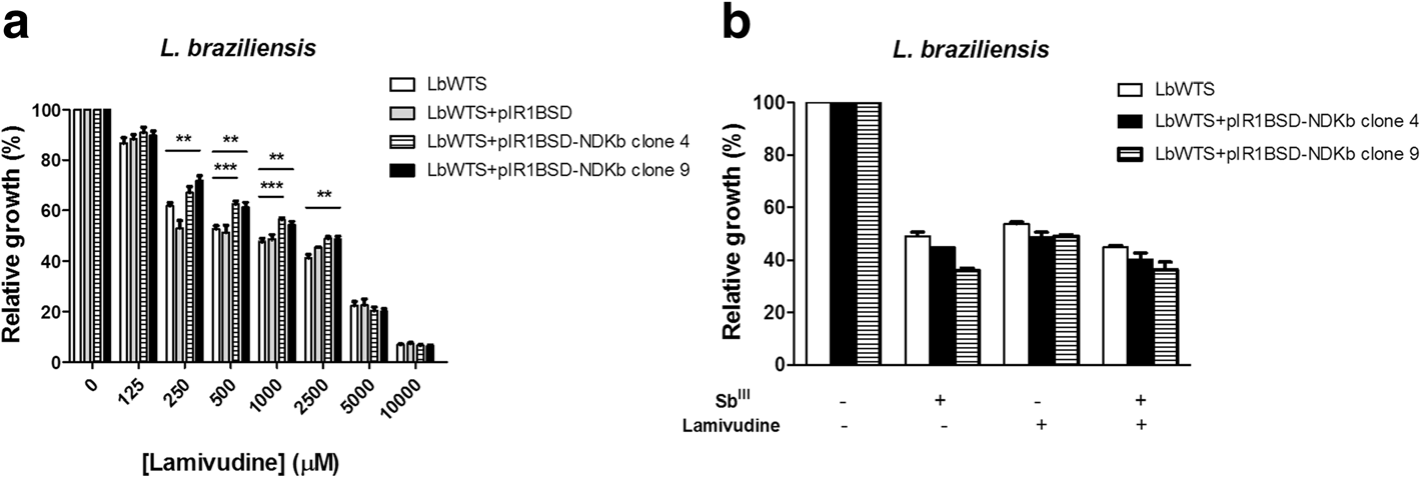
EC_50_ of lamivudine for wild-type and NDKb-overexpressing *L. braziliensis* lines (**a**) and effect of lamivudine on the growth of *L. braziliensis* lines upon Sb^III^ exposure (**b**). Parasites were incubated in M199 medium in the absence or presence of different concentrations of lamivudine (C_8_H_11_N_3_O_3_S) (125 to 10,000 μM). For competition assay, cells were exposed to the EC_50_ of Sb^III^ (7, 12 and 15 μM for the LbWTS and NDKb-overexpressing clones 4 and 9, respectively) and the EC_50_ of lamivudine (766, 2110 and 2014 μM for the LbWTS and NDKb-overexpressing clones 4 and 9, respectively) independently or combined, followed by incubation for 48 h. The percentage of relative growth was determined using a Z1 Coulter Counter. Mean values ± standard deviations from three independent experiments in triplicate are shown. Statistical analysis was carried out using Student’s *t*-test. Statistically different values are highlighted as follows: **P < 0.01; ***P < 0.001. Pairwise comparisons (**a**): **250 μM:** LbWTS *vs* LbNDKb clone 9 (t_(7)_ = 4.04, *P* = 0.0049); **500 μM:** LbWTS *vs* LbNDKb clone 4 (t_(9)_ = 5.27, *P* = 0.0005); LbWTS *vs* LbNDKb clone 9 (t_(9)_ = 3.74, *P* = 0.0046); **1000 μM:** LbWTS *vs* LbNDKb clone 4 (t_(10)_ = 5.67, *P* = 0.0002); LbWTS *vs* LbNDKb clone 9 (t_(10)_ = 3.52, *P* = 0.0055); **2500 μM:** LbWTS *vs* LbNDKb clone 4 (t_(8)_ = 5.03, *P* = 0.0010); LbWTS *vs* LbNDKb clone 9 (t_(8)_ = 3.91, *P* = 0.0045)

Initially, the amino acid sequence of NDKb (TriTrypDB accession number LbrM.32.3210) was used to search possible drugs against this enzyme in the DrugBank (www.drugbank.ca). Search results for the submitted sequence returned three antiviral agents: tenofovir (ID DB00300), lamivudine (ID DB00709) and adefovir dipivoxil (ID DB00718). These drugs are recommended in the chemotherapy of chronic hepatitis B (HBV), and the first two are also useful to treat HIV infection. Lamivudine is a synthetic nucleoside analogue and is phosphorylated intracellularly to its active 5′-triphosphate metabolite (lamivudine triphosphate - LTP), which is included into viral DNA by HIV reverse transcriptase and HBV polymerase, causing the ending of DNA chain (www.drugbank.ca/drugs/DB00709). We determined the lamivudine EC_50_ of wild-type *L. braziliensis* line and parasites transfected with the constructs pIR1BSD (empty vector) and pIR1BSD-NDKb. Our data showed that the lamivudine EC_50_ were 2.6-fold and 2.8-fold higher for NDKb-overexpressing clones 9 and 4 (2,014 and 2,110 μM, respectively) when compared to LbWTS (766 μM) (Fig. 4a) (Table 1), demonstrating that these clones are more resistant to lamivudine. Competition assay was carried out to analyze the combination of lamivudine and Sb^III^ treatment in WTS and NDKb-overexpressing *L. braziliensis* lines. Then, the parasites were incubated simultaneously with their respective EC_50_ for each drug (Table 1), and after 48 h the relative growth of the parasites was calculated. The combined effect of both compounds reduced by 55%, 59.7% and 63.7%, respectively, parasite numbers of LbWTS, LbNDKb clones 4 and 9, showing that the lamivudine did not increase the leishmanicidal activity of Sb^III^ (Fig. 4b).

### Effect of EF2K inhibitor on the growth of wild-type and EF2-overexpressing *L. braziliensis* lines

**Fig. 5:**
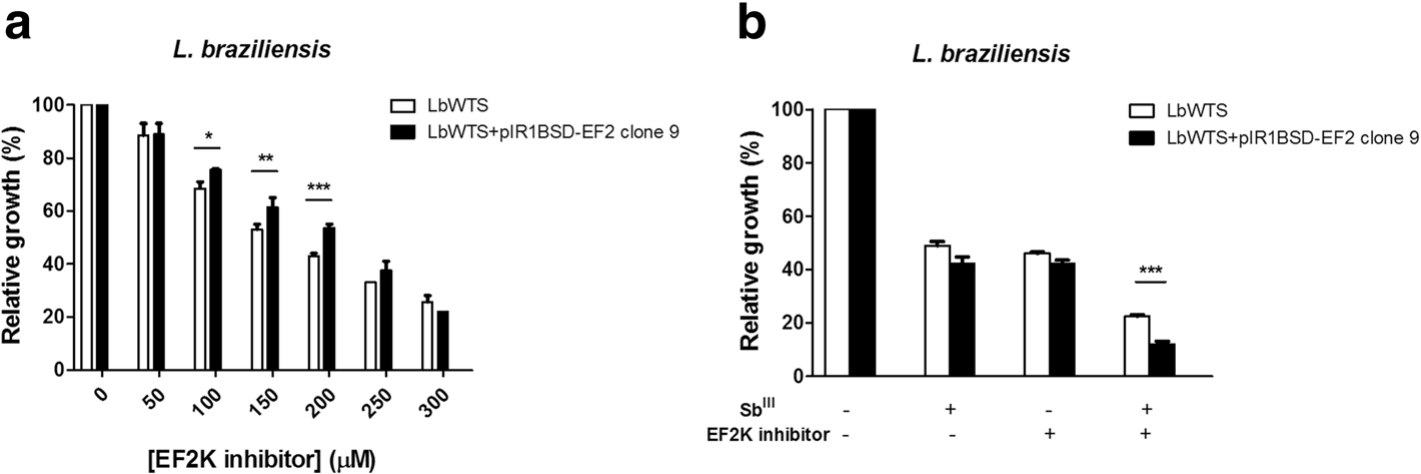
EC_50_ of elongation factor 2 kinase (EF2K) inhibitor for wild-type and EF2-overexpressing *L. braziliensis* lines (**a**) and effect of EF2K inhibitor on the growth of *L. braziliensis* lines upon Sb^III^ exposure (**b**). Parasites were incubated in M199 medium in the absence or presence of different concentrations of EF2K inhibitor (C_13_H_15_N_5_O_3_) (50 to 300 μM). For competition assay, cells were exposed to the EC_50_ of Sb^III^ (7 and 14 μM for the LbWTS and EF2-overexpressing clone 9, respectively) and the EC_50_ of EF2K inhibitor (173 and 211 μM for the LbWTS and EF2-overexpressing clone 9, respectively) independently or combined, followed by incubation for 48 h. The percentage of relative growth was determined using a Z1 Coulter Counter. Mean values ± standard deviations from three independent experiments in triplicate are shown. Statistical analysis was carried out using Student’s *t*-test. Statistically different values are highlighted as follows: *P < 0.05; **P < 0.01; ***P < 0.001. Pairwise comparisons (**a**): **100 μM:** t_(6)_ = 3.52, *P* = 0.0126; **150 μM:** t_(6)_ = 3.94, *P* = 0.0076; **200 μM:** t_(6)_ = 8.83, *P* = 0.0001. (**b**) **Sb** ^**III**^ **+ EF2K inhibitor:** t_(4)_ = 11.72, *P* = 0.0003

Elongation factor 2 kinase (EF2K), a calmodulin-dependent protein, binds numerous up-stream signals to the regulation of protein synthesis. Thus, EF2K phosphorylates EF2 and inhibits the function of this enzyme [39]. Chen et al. [40] showed that inhibition of EF2K by EF2K inhibitor decreases EF2 phosphorylation however it has little effect on proliferation in the cancer cells. Then, we tested the potential leishmanicidal effect of this inhibitor against wild-type and EF2-overexpressing *L. braziliensis* lines. Incubation of parasites with different concentrations of EF2K inhibitor revealed that the EC_50_ of this drug was 1.2-fold higher for LbEF2 clone 9 (211 μM) in comparison with LbWTS (173 μM) (Fig. 5a) (Table 1). This result shows that the overexpression of EF2 enzyme protects slightly *Leishmania* from lethal action of EF2K inhibitor. In addition, we evaluated the effect of EF2K inhibitor on the growth of *L. braziliensis* lines upon Sb^III^ exposure. Surprisingly, the combined treatment Sb^III^ with EF2K inhibitor enhanced the leishmanicidal activity against both *L. braziliensis* lines compared to those incubated with Sb^III^ or EF2K inhibitor alone (Fig. 5b). Additionally, this increased Sb^III^ susceptibility was higher in the EF2-overexpressing *L. braziliensis* clone 9 (88% growth inhibition) than in the wild-type *L. braziliensis* line (77.7% growth inhibition), suggesting a possible involvement of EF2 in this activity.

### Susceptibility of NDKb-overexpressing *L. braziliensis* lines to H_2_O_2_

*L. braziliensis* clonal lines overexpressing *NDKb* gene were submitted to susceptibility assays with H_2_O_2_ to evaluate the tolerance to oxidative stress generated by different concentrations of this compound. Our results demonstrated that LbWTS line showed an H_2_O_2_ EC_50_ of 213 μM, and the LbNDKb clones 4 and 9 displayed the same value of EC_50_ for H_2_O_2_, which was equal to 207 μM (Table 1). These data suggest that NDKb enzyme is not directly involved in the defense against oxidative stress in *L. braziliensis*.

## Discussion

In the absence of effective vaccine against leishmaniasis, the only way to treat and control all forms of the disease is through the use of chemotherapy. Pentavalent antimonials are considered one of the main options of treatment; however these drugs have several toxic side effects and high resistance rates. Drug resistance in leishmaniasis has a multifactorial origin, involving different factors related to host, parasite and drug [41]. Thus, the comprehension of resistance molecular mechanisms in *Leishmania* spp. is essential to identify novel drug targets to prevent or reverse such mechanisms. Recently, our phosphoproteomic study identified the proteins nucleoside diphosphate kinase b (NDKb) and elongation factor 2 (EF2) differently modulated in antimony-resistant *L. braziliensis* samples [34]. The results presented in this study corroborate with these data once we observed that the NDKb and EF2 proteins were more and less expressed, respectively, in the LbSbR line than the LbWTS pair. Literature data to date provide no functional analysis of these proteins in *Leishmania*. Therefore, overexpression of *NDKb* and *EF2* genes in New World *L. braziliensis* and *L. infantum* species were performed here to investigate the contribution of these genes in antimony resistance phenotype.

NDKb is a member of the NDK family which is implicated in multiple cellular processes [42]. NDKs have a key role in the purine metabolism in pathogenic parasites such as *Leishmania* spp. and *Trypanosoma* spp., which makes these enzymes potential targets for the development of new strategies for trypanosomiasis treatment [43]. NDK, which corresponds to Nm23 gene family in the human genome, can be considered a tumor metastasis suppressor [44, 45]. Furthermore, it has differential abilities to modulate tumorigenesis [46]. Here we observed that NDKb protein is more expressed in Sb^III^-resistant *L. braziliensis* and *L. infantum* lines than in the corresponding wild-type lines. Interestingly, proteomic analysis showed that the NDK enzyme was more abundant in a benznidazole-resistant *Trypanosoma cruzi* population [47]. In our study, transfection of the *NDKb* gene in wild-type *L. braziliensis* and *L. infantum* lines led to increase of NDKb protein levels in the transfected clones when compared to their parental counterparts, as demonstrated by Western blot results. Functional assays showed that *L. braziliensis* clonal lines overexpressing this enzyme are less susceptible to Sb^III^ in relation to untransfected parasites. Nevertheless, no difference was observed in the susceptibility to antimony between the wild-type and NDKb-overexpressing *L. infantum* lines, demonstrating that this gene does not alter the Sb^III^-resistance phenotype in this species.

In our search for possible inhibitors of NDKb enzyme, we found a drug known as lamivudine that is used in the chemotherapy of HIV and HBV diseases. According to DrugBank (www.drugbank.ca/drugs/DB00709), lamivudine is phosphorylated to active metabolites that compete for integration into DNA of the virus. These metabolites inhibit the HIV reverse transcriptase enzyme competitively and function as a terminator of DNA chain synthesis. The absence of a 3′-OH group in the incorporated nucleoside analogue stops the formation of the 5′ to 3′ phosphodiester linkage required for DNA chain elongation, and thereby, the growth of viral DNA is finished. Our study showed that overexpression of the NDKb enzyme confers resistance to lamivudine. The results propose that the parasites express the active form of this enzyme and that lamivudine probably prevents the transfer of γ-phosphoryl groups from NTP to NDP in the parasite. In addition, our results revealed that the combination lamivudine and Sb^III^ does not alter the leishmanicidal effect, suggesting that this combination is not a good strategy to be used in the leishmaniasis chemotherapy.

Attenuation of reactive oxygen species (ROS) production is an additional function proposed for secreted NDKs by pathogenic microorganisms [48]. Our results revealed that overexpression of NDKb enzyme did not alter the susceptibility of parasites to H_2_O_2_. These findings suggest that NDKb enzyme is not directly involved in the defense against oxidative stress in *L. braziliensis*.

Molecular mechanisms that regulate protein synthesis are essential for various biological phenomena. Protein translation is a process regulated at the initiation and elongation levels. There are diverse factors responsible for the regulation at translation elongation, but EF2 along EF1A are one of the most important enzymes which conduct the elongation cycle of protein synthesis in eukaryotic cells [49]. Our previous phosphoproteomic study demonstrated that EF2 protein presented lower abundance in Sb^III^-resistant *L. braziliensis* samples [34], suggesting that this protein was dephosphorylated (active state) to regulate the elongation of essential proteins which are crucial to maintain the antimony resistance phenotype. Other studies also found EF2 contributing to this phenotype in different *Leishmania* species. EF2 was found with higher abundance in Sb^V^-resistant *L. donovani* isolates [50], Sb^III^-resistant *L. panamensis* and *L. infantum* lines [33, 51]. However, according our previous results this protein probably might be dephosphorylated in these antimony-resistant parasites. Thus, these studies demonstrate that there are differences among *Leishmania* species, which can result in variations in the degree of phosphorylation or expression of the EF2 protein.

In this work, we transfected the *EF2* gene to analyze if the overexpression of this enzyme contributes for Sb^III^ resistance in *Leishmania* species. Western blot assays revealed that the expression level of this protein was higher in *L. braziliensis* and *L. infantum* lines overexpressing EF2 in comparison with their respective wild-type counterparts. Experiments of susceptibility to antimony showed that both *L. braziliensis* clones transfected with *EF2* gene were more resistant to Sb^III^ than the controls (wild-type and empty vector). On the other hand, the EF2-overexpressing *L. infantum* lines did not present difference in the susceptibility to Sb^III^ when compared to its parental line, showing that this gene is not relevant to Sb^III^ resistance in this *Leishmania* species. Overexpression of EF2 was observed in patients with lung adenocarcinoma [52] and related with reduced cell death after exposure to cumene hydroperoxide [53]. Kushawaha et al. [54] demonstrated that EF2, a Th1 stimulatory protein of *L. donovani*, produces strong IFN-γ and IL-12 response in cured *Leishmania*-infected patients/hamsters and confers considerable protection against experimental visceral leishmaniasis.

EF2 can be regulated through inhibitory phosphorylation at threonine 56 (T56) by EF2K [55]. Phosphorylation on T56 inactivates EF2 and it is the unique known regular EF2 functional alteration. Differently, EF2K suffers vast regulatory phosphorylations that permit distinct pathways to impact elongation [32]. Our results showed that EF2-overexpressing clone showed slight resistance to the EF2K inhibitor in comparison with the wild-type line. Surprisingly, this inhibitor increased the antileishmanial effect of Sb^III^, especially against EF2-overexpressing parasites. Chen et al. [40] showed that the concentrations of this inhibitor that effectively inhibited EF2 phosphorylation did not produce significant inhibition of cancer cell proliferation.

Our results demonstrated that both NDKb and EF2 proteins of *Leishmania* presented approximately 60% of identity with the mammal proteins, indicating a good degree of conservation between these proteins. In this way, further studies are needed to investigate the cytotoxicity of lamivudine and EF2K inhibitor against mammalian cells.

## Conclusions

Our findings represent the first study of *NDKb* and *EF2* genes overexpression in *Leishmania* species, demonstrating that these proteins are implicated in Sb^III^ resistance phenotype in *L. braziliensis*. Susceptibility assays showed that NDKb-overexpressing lines were more resistant to lamivudine, and EF2-overexpressing clone was moderately more resistant to EF2K inhibitor. In addition, our results suggest that the combined treatment EF2K inhibitor with Sb^III^ might be a good strategy to increase antileishmanial effect. Therefore, our data provided in this report bring new knowledges about resistance to Sb^III^ in *Leishmania* which can contribute to develop new strategies for leishmaniasis chemotherapy.

## Abbreviations

EF2: Elongation factor 2
EF2K: Elongation factor 2 kinase
Lb: *Leishmania* (*V.*) *braziliensis*
Li: *Leishmania* (*L.*) *infantum*
NDKb: Nucleoside diphosphate kinase b
Sb^III^: Potassium antimonyl tartrate
SbR: Sb^III^-resistant
WTS: Wild-type susceptible
